# Experimental study of hemodynamics in the circle of willis

**DOI:** 10.1186/1475-925X-14-S1-S10

**Published:** 2015-01-09

**Authors:** Guangyu Zhu, Qi Yuan, Jian Yang, Joon Hock Yeo

**Affiliations:** 1 School of Energy and Power Engineering, Xi'an Jiaotong University, Xi'an, 710049, Shaanxi, China; 2 Department of Radiology and Medical Imaging, the First Affiliated Hospital of Xi'an Jiaotong University, Xi'an, 710061, Shaanxi, China; 3 School of Mechanical and Aerospace Engineering, Nanyang Technological University, Singapore, 639798, Singapore

## Abstract

**Background:**

The Circle of Willis (CoW) is an important collateral pathway of the cerebral blood flow. An experimental study of the cerebral blood flow (CBF) distribution in different anatomical variations may help to a better understanding of the collateral mechanism of the CoW.

**Methods:**

An *in-vitro *test rig was developed to simulate the physiological cerebral blood flow in the CoW. Ten anatomical variations were considered in this study, include a set of different degrees of stenosis in L-ICA and L-ICA occlusion coexist with common anatomical variations. Volume flow rates of efferent arteries and pressure signals at the end of communicating arteries of each case were recorded. Physiological pressure waveforms were applied as inlet boundary condition.

**Results:**

In the development of L-ICA stenosis, the total CBF decreases with the increase of stenosis degree. The blood supply of ipsilateral middle cerebral artery (MCA) was affected most by the stenosis of L-ICA. Anterior communicating artery (ACoA) and ipsilateral posterior communicating artery (PCoA) function as important collateral pathways of cerebral collateral circulation when unilateral stenosis occurred. The blood supply of anterior cerebral circulation was compensated by the posterior cerebral circulation through ipsilateral PCoA when L-ICA stenosis degree is greater than 40% and the affected side was compensated immediately by the unaffected side through ACoA. Blood flow of the anterior circulation and the total CBF reached the minimum among all cases studied when L-ICA occlusion coexist with the absence of PCoA.

**Conclusion:**

The results demonstrated the flow distribution patterns of the CoW under anatomical variations and clarified the collateral mechanism of the CoW. The flow ACoA is the most sensitive indexes to the morphology change of ipsilateral ICA. The relative independence of the circulation in anterior and posterior sections of the CoW is not broken and the function of ipsilateral PCoA is not activated until a severe stenosis of unilateral ICA occurs. PCoA is the most important collateral pathway of the collateral circulation and the missing of PCoA has the highest risk of stroke when the ipsilateral ICA has severe stenosis. These findings may provide the basis for future therapeutic and diagnosis applications.

## Background

The Circle of Willis (CoW) is a ring like artery structure located at the bottom of brain. This structure provides important collateral circulation paths to maintain the sufficient blood supply, especially when stenosis or surgical clamping of the cerebral arteries happens. These collaterals consist of cross-flow through the anterior communicating artery toward the ipsilateral sphere of the ICA lesion, posterior to anterior flow through the ipsilateral posterior communicating artery, or both these systems [1-4].

**Figure 1:**
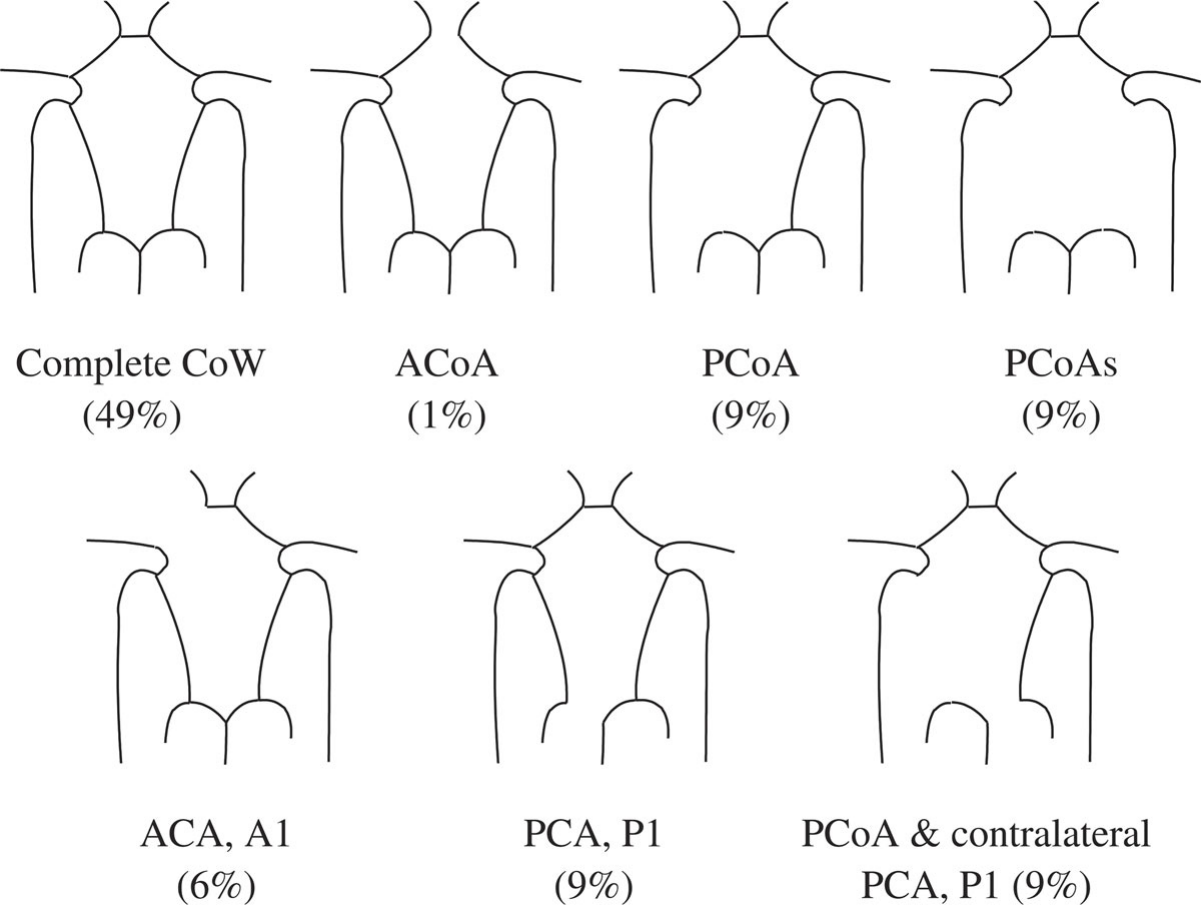
**Some common variations of the Circle of Willis **[48].

Unfortunately, congenital incompleteness of CoW, such as the lack of anterior communicating artery, unilateral or bilateral PCoA, is found in 50%-60% of the population [5-7]. Other anatomical variations, including fused vessels, string-like vessels and the presence of extra vessels, are also not uncommon (Figure 1)[8]. Such variations undermine the compensational capability of the artery, and can cause undesirable clinical consequences, including ischemia stroke and transient ischemia attack (TIA). When either anatomical variations coexist with ICA stenosis or surgical clamping, the risk would be even higher. Therefore, it is important to be able to assess the blood distribution and collateral flow patterns of the CoW under various anatomical and pathological situations to help understanding the mechanism of stroke, planning the clinical surgery and diagnosis of early stage stroke.

In the literature, there are many papers proposing various methods for identifying the collateral flow patterns in complete and incomplete CoW. Clinically, the using of Transcranial Doppler (TCD) [9-20], Magnetic Resonance Angiography (MRA)[13,15,17,19,21-26] and Computed Tomography Angiography (CTA)[22,27-29] provide the *in-vivo *perspective of the role of the CoW in the collateral flow in anatomical variations. Numerically, one-dimensional [30-36], two-dimensional [37,38] and three-dimensional [31,34,39-43] models of the CoW were developed, flows in the CoW with common anatomical variations were investigated.

In contrast to a host of clinical and numerical studies, however, only limited *in-vitro *experimental studies were reported in open literature. Cieslicki et al [44] carried out *in-vitro *investigation of flow distribution in the CoW under four extreme cases, including complete CoW, unilateral ICA occlusion, complete occlusion of the vertebral-basilar system and bilateral ICA occlusion. Fahy et al [45] observed the flow distribution in three different patient specific model of CoW and assessed the risk factor brought by anatomical variations, including missing of unilateral PCoA, unilateral pre-communicating part of the posterior cerebral artery, unilateral pre-communicating part of the anterior cerebral artery, bilateral PCoAs. In addition to limited quantity, there is a lack of *in-vitro *investigation that reveals the flow distribution in the development of ICA stenosis and ICA occlusion coexist with anatomical variations.

Moreover, details of the collateral mechanism of complete circle and incomplete circle are remaining debatable. Conflicting results have been reported on the flow patterns in the communicating arteries and importance of the collateral pathways. For the complete CoW, some *in-vitro *[46] and *in-vivo *[17] studies mentioned there are no flow across ACoA in a complete CoW, this phenomenon was also supported by numerical simulations [32,33,47]; Other numerical [48,49] and * in-vitro *[45] studies, in contrast, suggested flow across ACoA observed in complete circle of Willis under symmetrical boundary conditions. When unilateral ICA stenosis or occlusion occurred, Hartamp et al.[50], Miralles et al.[51] and Hoksbergen et al. [16] concluded that ACoA plays a more important role than PCoA in patients with severe ICA stenosis; Results from other studies suggested the fetal type or missing of ipsilateral PCoA is the only risk factor for ischemic cerebral symbols [4,38,51].

Motivated by the lacking of extensive *in-vitro *experimental studies and the conflicting research findings, an *in-vitro *experimental study focus on the collateral flow patterns in the CoW was carried out. A series of different degree of stenosis in unilateral ICA and unilateral ICA occlusion coexist with anatomical variations were simulated in this study. The CBF distributions in efferent arteries, cross flow in communicating arteries and collateral circulation mechanism of the CoW were investigated.

## Methods

### Model preparation

In previous studies, various techniques were used to measure the geometry of the CoW, including CTA imaging, MRI and direct measure of cerebral vessels. Due to individual differences, the dimensions of each artery segment are varied from case to case. In this study, average length and diameter from previous studies were used in this study to achieve a universal model [33,36,44,47,52]. The dimensions of each segment are listed in Table 1 and a physical model based on these dimensions was constructed. To simplify the model, each vessel is represented by a single silicon tube of constant radius. This simplification is acceptable because the length of every segment is relatively short and the conicity index is relatively small at cerebral artery site.

**Table 1:** Diameters and length of vessels.

	Segment Name	Diameter (mm)	Length (mm)
ACA-A1	Proximal Anterior Cerebral Artery	2.5	20
ACA-A2	Distal Anterior Cerebral Artery	3.0	250
ACoA	Anterior Communicating Artery	2.0	3
BA	Basilar Artery	5.0	30
ICA	Internal Carotid Artery	4.5	250
PCA	Posterior Cerebral Artery	3.0	250
PCoA	Posterior Communicating Artery	1.8	20
VA	Vertebral Artery	3.0	250

To simulate the different degrees of ICA stenosis, a set of connectors with different flow tunnel diameter was prepared. All connectors were made by copper cylinders of a length of 20 mm and an outer diameter of 4.5 mm. Flow tunnels with different diameters were drilled at the centre of the cross section along the axial direction to simulate different degrees of stenosis of ICA. The degree of stenosis is defined as the ratio of inner diameter of stenosed artery over original inner diameter. L-ICA occlusion was simulated by clamping the tube using a hemostat.

### Physiological flow system

**Figure 2:**
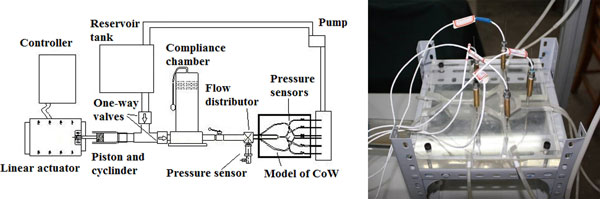
**Schematic of experimental test rig**.

The schematic diagram of the patented (ZL 200910021375.5) test rig is shown in Figure 2. A linear actuator (LMS 50, PBA System Pte. Ltd., Singapore) controlled by a programmable servo (Cornet COR 5/230, Elmo Motion Control Ltd., Israel) was used to drive the piston to generate pulsatile pressure waveforms. Function as mitral valve and aortic valve to prevent back flow, two one-way valves were connected to reservoir tank and compliance chamber, respectively. The effect of artery compliance was simulated by imposing an air chamber which air volume is adjustable. A flow distributor after the compliance chamber separated the flow into four branches and connected to the four afferent arteries of the CoW model.

**Figure 3:**
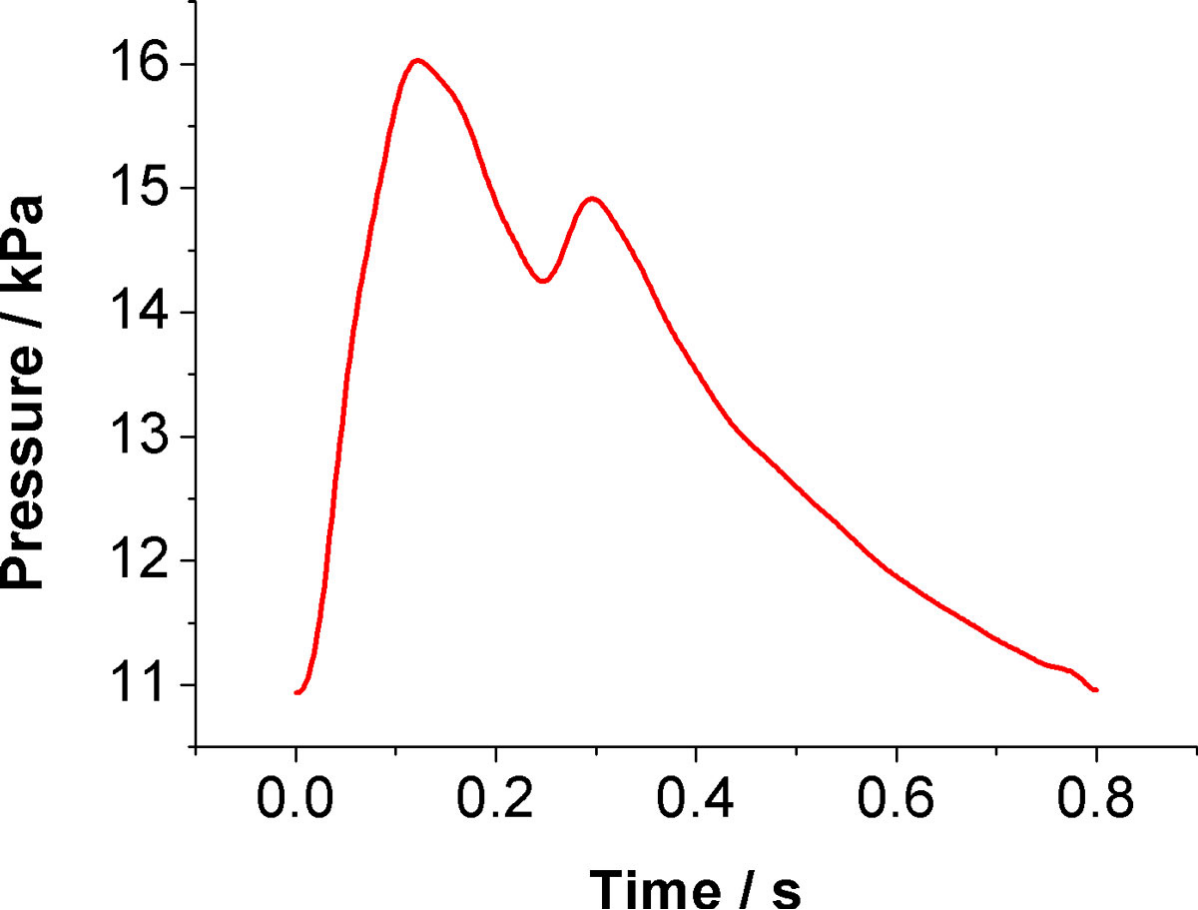
**Inlet pressure waveform**.

A physiological pulsatile pressure waveform for the bilateral ICA and VA were applied as inlet boundary condition (Figure 3). The systolic pressure, diastolic pressure and average pressure are 16.1 kPa (120.7 mmHg), 10.97 kPa (82 mmHg) and 12.7 kPa (95 mmHg), respectively [44,45,53]. The inlet pressure was measured by using dynamic pressure transducer (ATM231, Sensoren Transmitter Systeme GmbH, Sindelfingen, Germany). Six micro pressure transducers (XCQ-062, Kulite Semiconductor Products Inc., Ridgefield, NJ, USA) were installed at six bifurcation sites of the model, include the the bifurcation at the junction of bilateral PCA and PCoA, the bifurcation at the junction of bilateral MCA and PCoA and the bifurcation at the junction of bilateral ACA and ACoA. Pressure signals at these sites were recorded by a data acquisition system (Vision XP, LDS Test and Measurement GmbH, Ismaning, Germany).

Adjustable resistors were connected to the end of efferent arteries. Six beakers were placed at ends of the efferent arteries, and volume of fluid flows out from each efferent artery was measured periodically, to calculate the total mean volume flow rates and the flow rate of each efferent artery. The fluid collecting system simultaneously collected outflows from the CoW and distributes the fluid into six beakers so that the mean flow rate can be measured. The total flow rate of the complete CoW was set at physiological level of 760 ml·min^-1^, and the flow rate ratio between VAs and ICAs was 30:70. The cardiac cycle was set to 0.8 s (75 beats·min^-1^).

All devices were turned on 30 minutes before data collection for warm up and reach a stable condition. Measurements were collected for 100 consecutive cardiac cycles, and were repeated three times for each cases mentioned above. In the complete CoW, the pressure waveforms measured over 100 cycles in L-ICA was within 0.1<% for peak systolic pressure and peak diastolic pressure respectively, shows an excellent repeatability between cycles. The average pressure difference between L-ICA and R-ICA of the complete CoW at systolic peak is less than 0.8%.

### Working fluid

Blood is a non-Newtonian multiphase fluid with shear thinning properties. However, most researchers have considered blood as a Newtonian fluid except when they study the blood flow in medium to large arteries [54,55]. In this study, blood was assumed as Newtonian fluid. The working fluid applied here is a 40% aqueous solution of glycerin (volume ratio), the viscosity and density of which is 3.5 mPa·s (DV-III+, Brookfield, USA) and 1056 kg·m^-3^, respectively.

### Experimental methods

To investigate the blood flow distribution in the CoW under different ICA stenosis degrees, a series of pathological variations were considered, including:

1) Complete circle;

2) Different stenosis degrees of L-ICA (40%, 50%, 60%, 75%, and 87.5%) and L-ICA complete occlusion;

3) L-ICA occlusion coexist with the absence of communication arteries (R-PCoA, ACoA and L-PCoA).

Flow rates of above conditions were recorded and analysed.

To investigate the collateral mechanism of the CoW, pressure at both ends of each communicating artery (ACoA, L-PCoA and R-PCoA) under different L-ICA stenosis degrees was recorded, and this also provide indication of the flow direction.

## Results

### Stenosis at L-ICA versus volume flow rate

**Figure 4:**
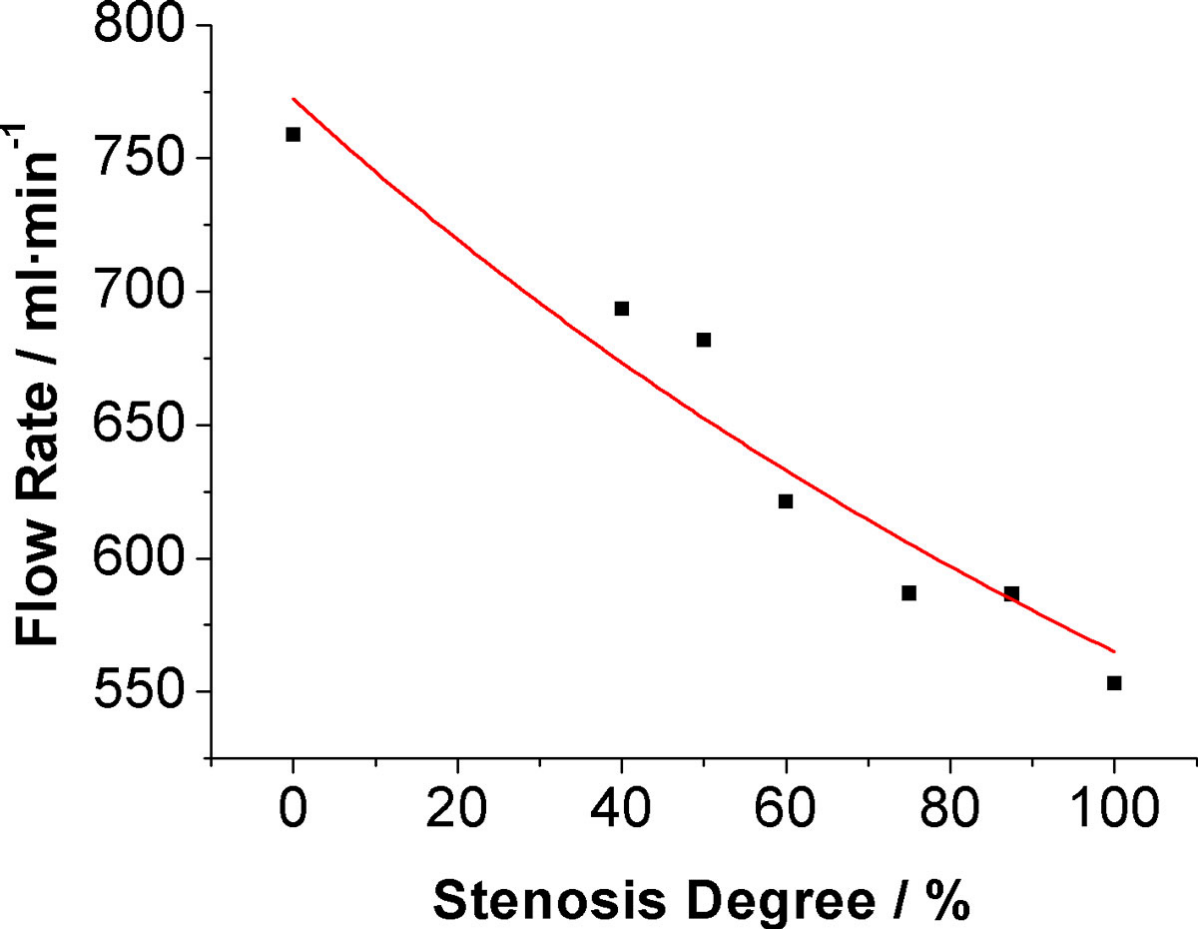
**Total flow rate under different L-ICA stenosis degree**.

The total volume flow rates of CoW under different L-ICA stenosis degrees are illustrated in Figure 4. Detailed data were shown in Table 2. When a stenosis was introduced to the L-ICA, the total flow rate was directly affected. As the stenosis degree increased, the flow rate decreased continuously. When L-ICA was completely occluded, the total flow rate decreased by about 205 ml·min^-1^. This is about 27% of the normal flow rate. In complete CoW, the distribution of flow to anterior, middle and posterior cerebral artery was accounts for 21.19%, 51.49% and 27.27% of total flow rate, respectively.

**Table 2:** Total flow rates under different stenosis degree of L-ICA.

Stenosis degree (%)	Total flow rate (ml·min^-1^)	Percentage change (%)
0	758.93	0
40	693.58	-8.61
50	681.89	-10.15
60	621.32	-18.13
75	586.92	-22.66
87.5	585.56	-22.84
100	553.21	-27.10

**Figure 5:**
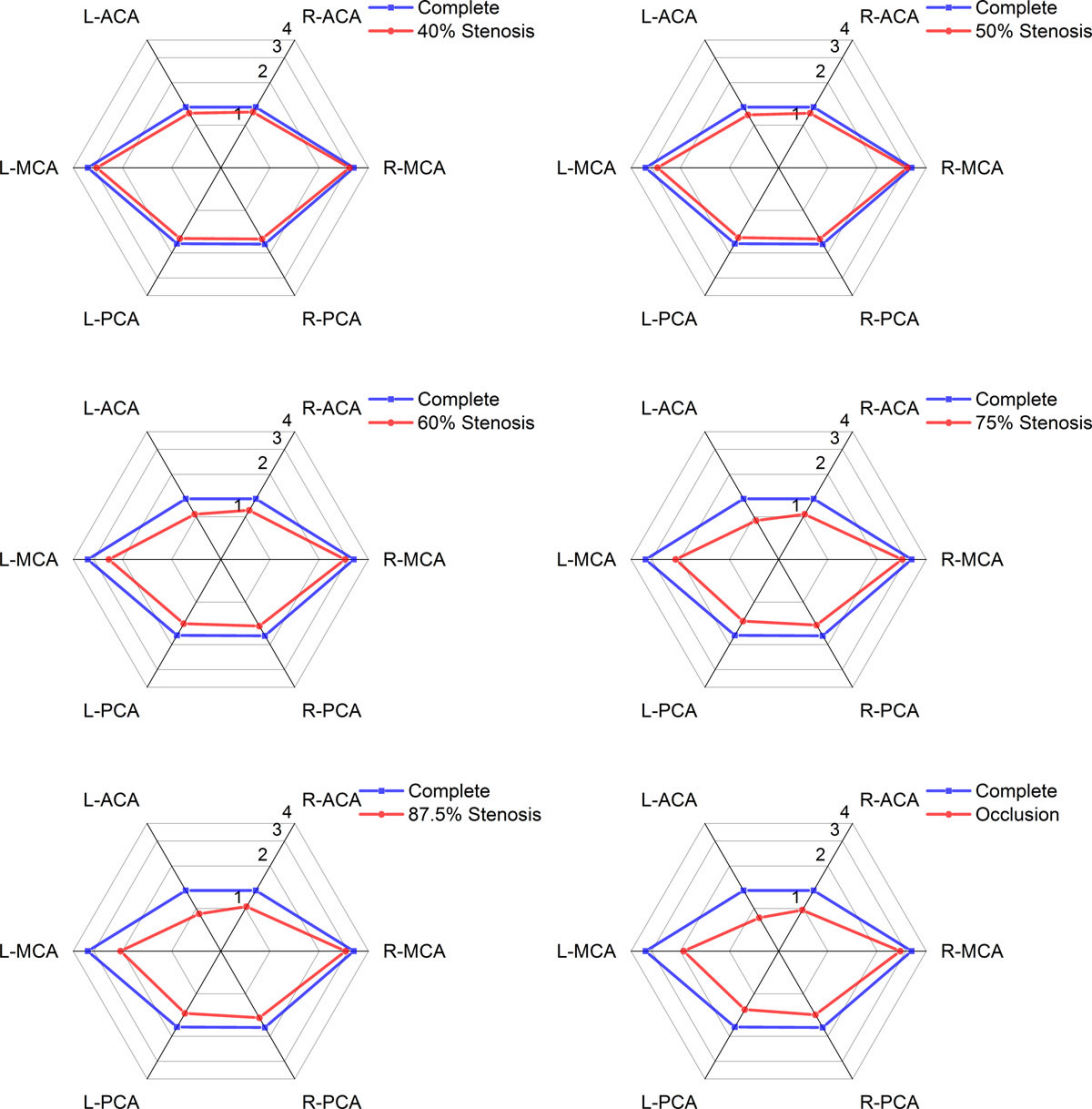
**Flow rates of efferent arteries under different L-ICA stenosis degree (ml·s^-1^)**.

Figure 5 is a schematic illustrating the change of flow rates in efferent arteries (ACA, MCA and PCA) under different L-ICA stenosis degrees. The specific flow rates of efferent arteries are shown in Table 3.

**Table 3:** Flow rates of efferent arteries under different stenosis degree of L-ICA.

Stenosis degree (%)	Flow rate in efferent arteries (ml·s^-1^)					
	
	R-ACA	R-MCA	R-PCA	L-PCA	L-MCA	L-ACA
0	1.34	3.26	1.73	1.72	3.26	1.34
40	1.24	3.06	1.59	1.58	2.87	1.22
50	1.22	3.06	1.59	1.56	2.75	1.18
60	1.11	2.88	1.48	1.42	2.42	1.04
75	1.04	2.84	1.46	1.36	2.13	0.94
87.5	1.03	2.90	1.48	1.38	2.05	0.92
100	0.97	2.78	1.41	1.30	1.90	0.86

There was an immediate reduction in blood flow through all of the efferent arteries when stenosis occurred, and the flow rates of all efferent arteries were decreased with the increasing of the stenosis degree of L-ICA. The three efferent arteries ipsilateral to the stenosis show the greatest relative reduction. When L-ICA was occluded, flow rate in the affected side of the ACA, MCA and PCA was 36.0%, 40.7% and 24.8% lower than normal conditions, respectively. The flow rate in the healthy side of the ACA, MCA and PCA was only 27.3%, 13.3% and 17.3% lower than normal conditions, respectively. This observation suggests that the L-MCA is affected most by the stenosis of L-ICA.

Under mild to medium degree of stenosis (<50%), the volume flow rates in the ACA and PCA were decreased synchronously, and no obvious differences between bilateral branches were observed. When the stenosis degree is greater than 75%, the result shows that the flow rates in bilateral ACA and PCA to become off-balance. This is an indication that the symmetry of the CoW is compromised. Compare with the ACA and PCA, the MCA is more sensitive to the morphology change of the ICA, see Table 3 and 4. The imbalanced flow between the bilateral MCA that appeared under mild stenosis and the difference in flow rates between the L-MCA and R-MCA increases continuously with the degree of stenosis.

**Table 4:** Percentage change in flow rates of efferent arteries under different stenosis degree of L-ICA.

Stenosis degree (%)	Percentage change in flow rate (%)					
	
	R-ACA	R-MCA	R-PCA	L-PCA	L-MCA	L-ACA
0	0.00	0.00	0.00	0.00	0.00	0.00
40	-7.69	-5.98	-7.95	-8.00	-11.97	-9.39
50	-9.39	-5.98	-7.95	-9.33	-15.49	-11.96
60	-17.09	-11.62	-14.57	-17.33	-25.70	-22.22
75	-22.22	-12.68	-15.89	-20.66	-34.51	-29.91
87.5	-23.07	-10.92	-14.57	-20.00	-36.97	-31.61
100	-27.34	-14.79	-18.55	-24.67	-41.55	-35.89

The percentage change in flow rate of efferent arteries under different stenosis degree in Table 4 consistently shows the highest percentage change occurring in the L-MAC.

### Flow direction in communicating arteries

**Figure 6:**
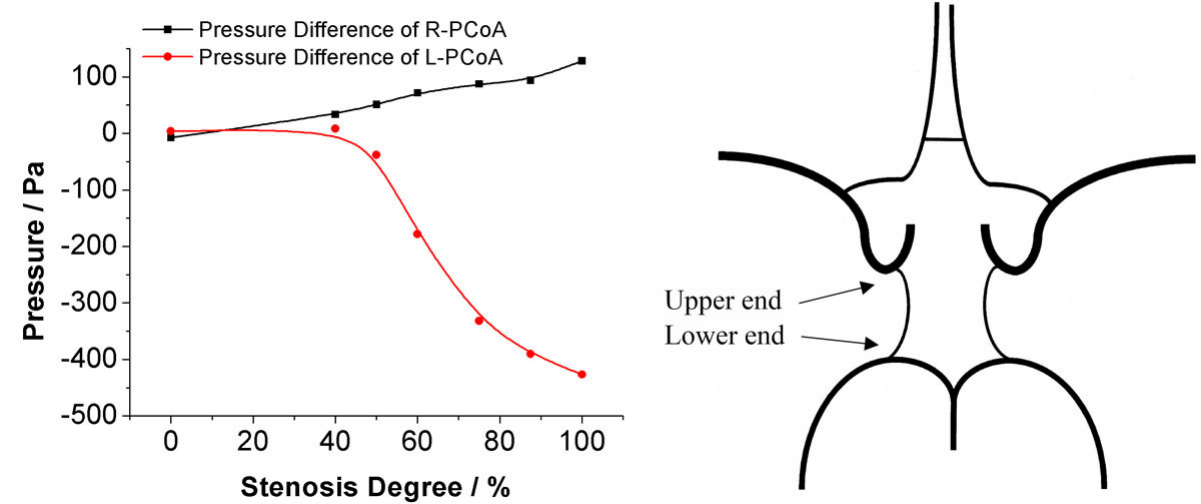
**Pressure difference of PCoA**.

To investigate the flow direction in communicating arteries, pressure signals were recorded at both ends of each communicating artery. Figure 6 shows the pressure difference between the upper end and lower end of L-PCoA and R-PCoA. Here, the upper end of PCoA is defined as the junction with MCA, and the lower end is the junction with PCA; the pressure difference of PCoA is the pressure at the upper end minus the pressure at the lower end. When no stenosis occurred, the pressure difference at both ends of the PCoA is near zero. Blood from VAs and ICAs just supply their own perfusion area and there is no blood flow through the PCoA. The pressure difference at the L-PCoA is found decreased sharply when the L-ICA stenosis degree is greater than 40%. This phenomenon can be explained as blood flow was drawing away from the PCA, and flows from posterior to anterior. In contrast, contralateral PCoA is virtually unaffected, the pressure difference at R-PCoA increases slightly during the increase of L-ICA stenosis rate.

**Figure 7:**
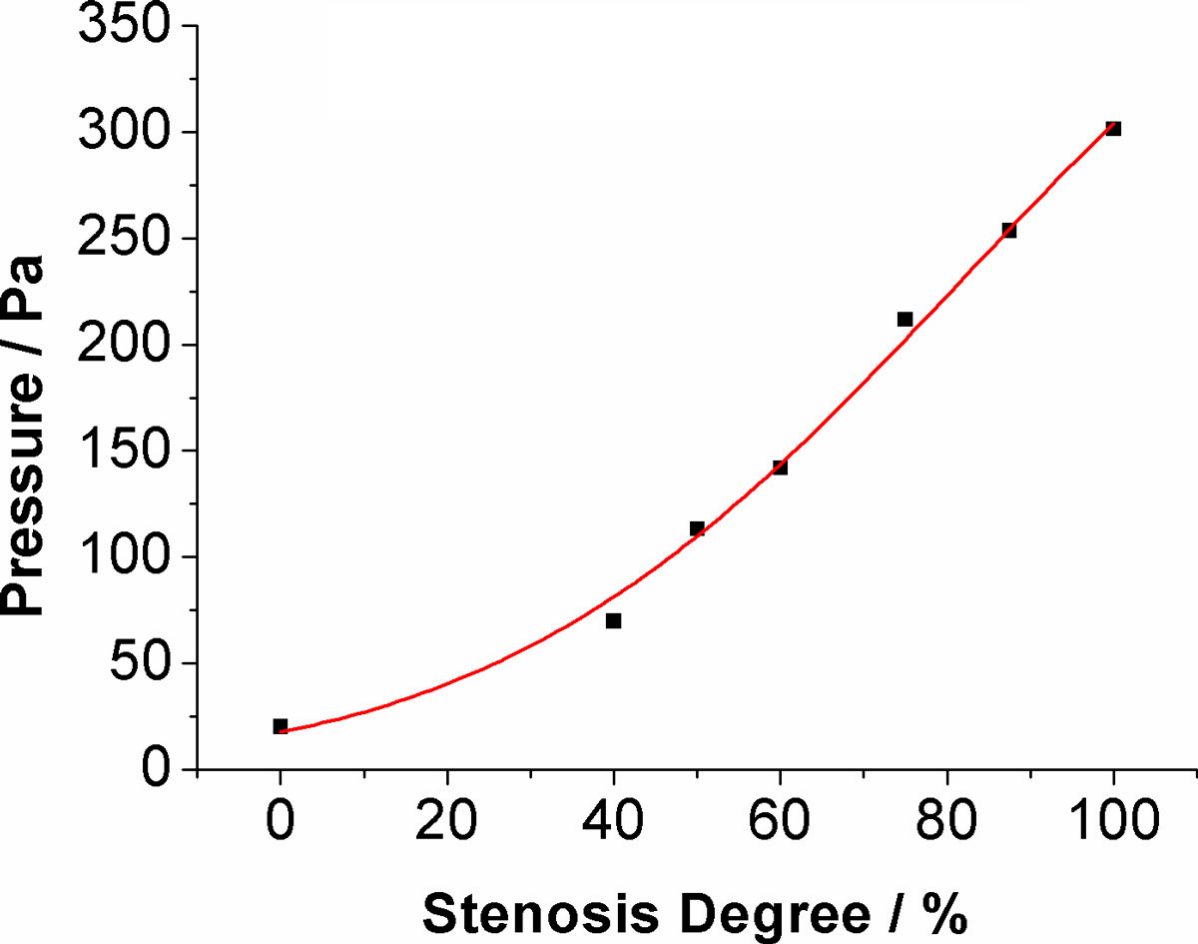
**Pressure difference of ACoA**.

Figure 7 shows the pressure difference of ACoA. This is the pressure at the right end of the ACoA minus the pressure at the left end of the ACoA. Under normal conditions, the pressure difference is near zero, which means no blood flow through ACoA. As the increase of L-ICA stenosis increases, the pressure difference also increases, the blood flow through ACoA from right to left.

### Flow distribution versus anatomical variations

In addition to 1) the complete CoW and 2) L-ICA occlusion, three anatomical variations were also investigated:

3) L-ICA occlusion coexist with absence of contralateral PCoA,

4) L-ICA occlusion coexist with absence of ACoA,

5) L-ICA occlusion coexist with absence of ipsilateral PCoA.

**Figure 8:**
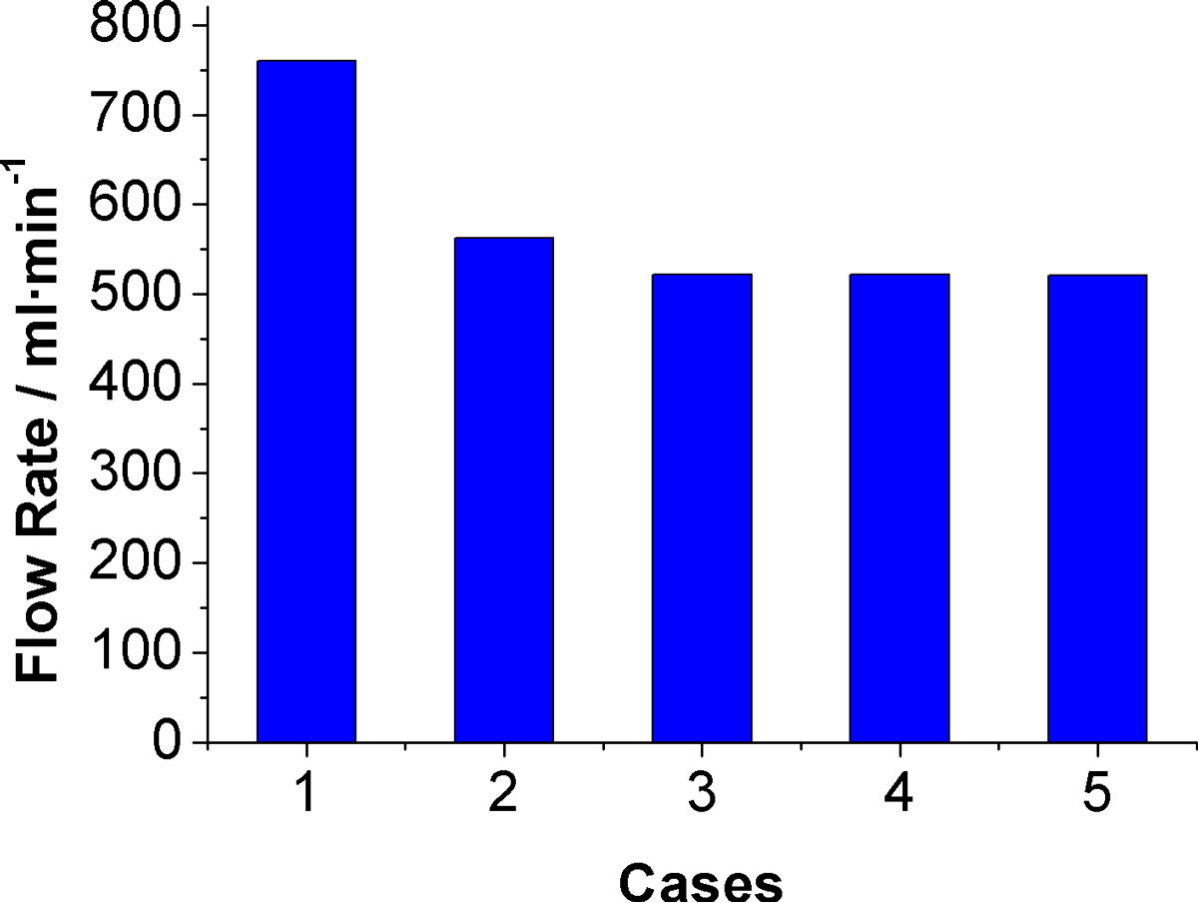
**Total flow rates under different variations**.

The total volume flow rate of case (3) to (5) was 31.3%, 31.3%, 31.4% lower than normal flow rate (case (1)) respectively, and about 7.1% lower than in case (2) where only occlusion occurred at the L-ICA (Figure 8).

**Figure 9:**
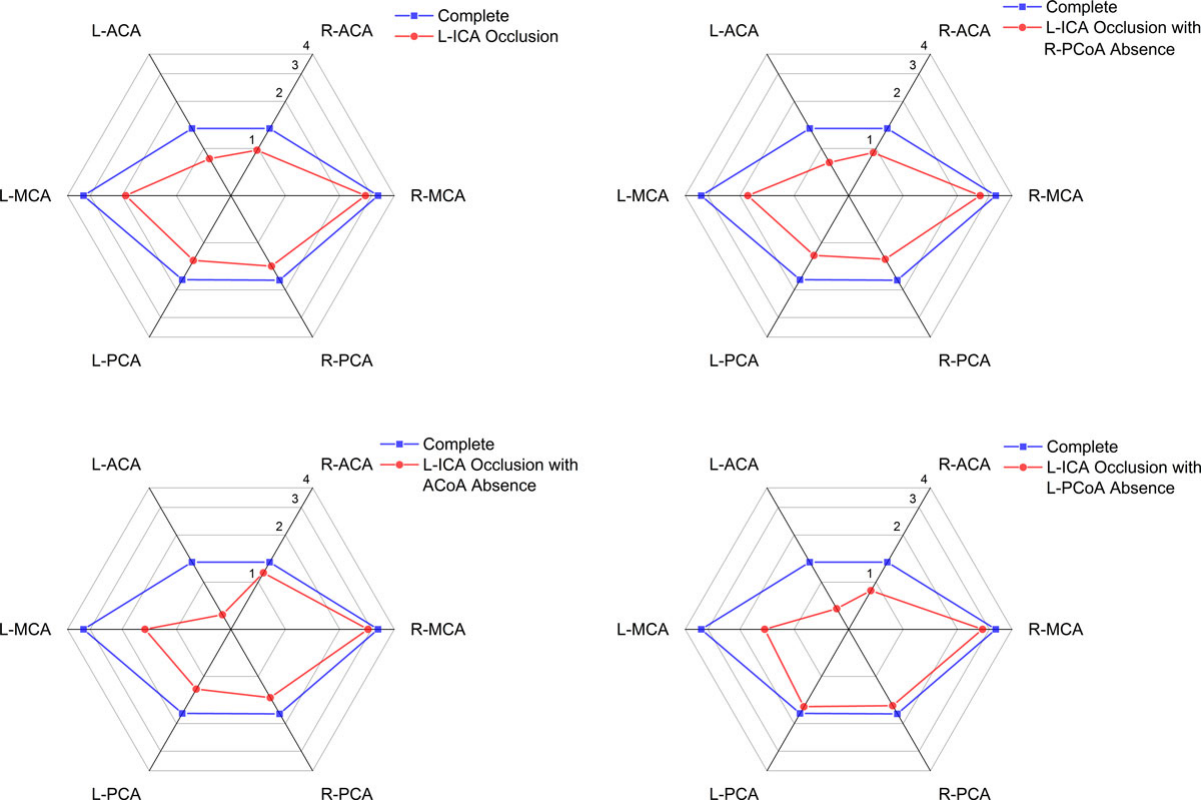
**Flow rates of efferent arteries under different variations (ml·s^-1^)**.

The volume flow rates of efferent arteries are shown in Figure 9, detailed data are listed in Table 5. When contralateral PCoA absent, no obvious change of flow rate in efferent arteries were observed except R-PCA, which decreased 10% compare with case (2).

**Table 5:** Flow rates of efferent arteries under the absence of communicating arteries.

Case	Flow rate in efferent arteries (ml·s^-1^)					
	
	R-ACA	R-MCA	R-PCA	L-PCA	L-MCA	L-ACA
1	1.34	3.26	1.73	1.72	3.26	1.34
2	0.97	2.78	1.41	1.30	1.90	0.86
3	0.94	2.67	1.27	1.20	1.80	0.81
4	1.15	2.87	1.36	1.20	1.49	0.62
5	0.88	2.75	1.54	1.56	1.45	0.68

Absence of ACoA coexist with occlusion of L-ICA is the most critical situation to ipsilateral ACA among all cases investigated. The flow rate in ACA ipsilateral to L-ICA is 28.1% lower than that in case (2). The absence of ACoA also decreasing the flow through ipsilateral MCA, which is 21.5% lower compare with case (2). Meanwhile, flow rate of MCA and PCA contralateral to L-ICA have no significant change compare with case (2). Flow rate in contralateral ACA increased 18.6% compare with case (2) and only 14.18% lower than complete CoW (case (1)).

In case (5), total CBF, flow rate in L-MCA and flow rate in R-ACA reached their lowest value compare with that of all cases. The flow rate in L-MCA and R-ACA is 23.43% and 9.28% lower than case (2), respectively. Flow rates in bilateral PCA are highest among case (2) to (5).

## Discussion

*In-vitro *experimental study were carried out in present study to investigate the CBF distributions in efferent arteries, cross flow in communicating arteries and collateral circulation mechanism of the CoW. There are many factors that can influence the distributions of flow rates in the CoW. In the present study, neither the auto-regulation mechanism of peripheral arterial beds nor the vessel elastic was considered. As mentioned, the aim of this study is not to create a model of the CoW that can simulate all physiological factors of a real circulation system, but simply to investigate the impact of structural variation on the flow distribution in the CoW and the basic collateral circulation mechanism.

In developing the model of the CoW, validation is of the utmost importance if the model results are to be trusted. Due to the lack of clinical data that cover all cases studied in this paper, a comparison based on the complete CoW was considered. The flow rates of efferent arteries were compared between this study and previous clinical measurements.

Table 6 listed the results from phase-contrast MRI measurements.

**Table 6:** Comparison of flow rates of efferent arteries with previous *in-vivo* measurements.

Study	Volume flow rate (ml·s^-1^)		
	
	ACAs	MCAs	PCAs
Current model (Complete CoW)	2.68	6.52	3.45
Enzmann et al. [57]	2.72 ± 0.35	3.92 ± 0.24	1.73 ± 0.14
Ooij et al. [58]^1,*^	3.0 ± 0.80	6.2 ± 1.60	2.0 ± 0.60
Ooij et al. [58]^2,*^	3.0 ± 0.80	4.8 ± 0.80	2.0 ± 0.60
Zhao et al. [59]	2.75 ± 0.90	4.92 ± 0.97	2.15 ± 0.47

*The data provided in original paper was flow rates in unilateral arteries. For comparison, data presented here were doubled.

1. Results from 7T scanning, 0.8 mm resolution

2. Results from 7T scanning, 0.5 mm resoulution

The flow rates in ICAs and ACAs agree well with *in-vivo *measurements data. The flow rates in MCAs, BA and most noticeably the PCAs, however, appear to slightly larger than *in-vivo *measurements. According to the simplify of current model, this phenomenon could be explained as the absence of branch arteries of ICAs and BA, include the ophthalmic and anterior choroidal arteries, may cause the raise of flow through MCAs and PCAs [47]. Further comparison was made between current results and previous numerical model [47] and *in-vitro *study [44], specific data were listed in Table 7.

**Table 7:** Comparison of flow rates of efferent arteries with previous numerical and *in-vitro* studies.

Study	Volume flow rate (ml·s^-1^)		
	
	ACAs	MCAs	PCAs
Current model (Complete CoW)	2.68	6.52	3.45
Moore et al. [47]	2.78	5.56	4.16
Cieslicki et al. [44]	3.1	4.85	3.76

These data presented in previous *in-vivo*, numerical and *in-vitro *studies are useful validations to show current *in-vitro *results are within acceptable physiological ranges. Therefore, the results in the study were convincing to provide information of CBF distribution and collateral circulation mechanism under such situations.

Notable decrease of flow rate in ACA and MCA was observed in the results, which indicate that the anterior circulation was affected most by ICA stenosis. When the stenosis degree of unilateral ICA increased from 0% to 100%, the flow rate in ipsilateral ACA and MCA decreased 35.89% and 41.55% respectively. These values can be validated in the numerical study of Zhang et al [32]. Flow rates in PCA is less affected by unilateral ICA stenosis. This may due to the blood flow of posterior circulation are mainly provided by VA-BA system.

The pressure signal provides more information about the collateral compensation mechanism of the CoW. In complete CoW, the pressure difference between both ends of ACoA is almost zero, which indicated there is no blood flow through ACoA and the left and right circulation is relatively independent when the CoW is complete. This finding was in contrast to some numerical [48,49] and in-vitro [45] studies, but it was in agreement other *in-vitro *[46], *in-vivo *[17] and numerical [30,47] studies. Uni-directional cross-flows occurs simultaneously in ACoA when stenosis induced to L-ICA. The blood flows through ACoA from the healthy side of the CoW to the affected side. As PCoA, the pressure difference of both PCoAs were near zero, which suggest there are almost no flow through PCoA in the complete CoW. When stenosis in unilateral ICA occurred, the pressure signals indicate that ipsilateral PCoA provides an important collateral pathway in which the blood flow toward anterior circulation. This observation is supported by clinical TCD measurement [17]. The collateral compensation function of ipsilateral PCoA will be fully activated when the stenosis degree at L-ICA is greater than 40%. Contralateral PCoA, however, only provides limited support to the compensation blood supply as the pressure difference is very small even L-ICA was complete occlude.

The role of PCoA and ACoA in collateral circulation was further revealed when take the anatomical variations into consideration. When the L-ICA occlusion coexist with the absence of ACoA, minimum flow rate in L-ACA was observed among all cases studied. This observation indicate that ACoA is an utmost important collateral path for ACA at affected side. For the whole anterior circulation and the total CBF supply, however, PCoA ipsilateral to stenosed ICA plays a more important role than ACoA in the collateral circulation. This conclusion is based on the observation that the flow rates in anterior circulation and the total CBF supply reached their minimum value when L-ICA occlusion coexist with the absence of ipsilateral PCoA. These observations are well agreed with clinical results [56].

This study had several limitations. Some vessels of the CoW, include ophthalmic artery, choroidal arteries and superior cerebellar arteries were simplified. As discussed before, the simplification would influence the flow distribution in the CoW. Moreover, flow visualization in current model is impossible. This drawback prevent us from access more detailed flow parameters, such as flow pattern and wall shear stress, which may help us have a further understanding of the hemodynamics of the CoW under different anatomical configurations. To access the wave propagation in the CoW, the vessel elasticity also need to be considered in future work.

## Conclusions

Flow distribution patterns of the CoW under anatomical variations and the collateral mechanism of the CoW were investigated and clarified in present work. Experiment showed that PCoA is the most important collateral pathway in cerebral collateral circulation. The anatomical variation missing PCoA has the highest risk of TIA and cerebral stroke when the ipsilateral ICA has severe stenosis. In addition, the anterior circulation and posterior circulation of the CoW was found to be independent to each other until the ICA stenosis become severe. Moreover, we found that the collateral capacity of PCoA won't be fully activated until ICA stenosis degree is greater than 40%. Further more, the results indicate that the flow in ACoA minght be used as a criterion of morphology change of ICA. These findings may potentially be used to enhance our understanding of the hemodynamics in the CoW and eventually lead to future therapeutic and diagnosis applications.

## Competing interests

The authors declare that they have no competing interests.

## Authors' contributions

GYZ carried out the experimental study, participated in the sequence alignment and drafted the manuscript. QY participated in the design of this study and sequence alignment as supervisor. JY participated in the sequence alignment. JHY participated in the design of the study and helped to draft the manuscript.

## Declarations

Publication charges for this article have been funded by Interdisciplinary Research Foundation of Xi'an Jiaotong University (XKJC2013013).

This article has been published as part of *BioMedical Engineering OnLine *Volume 14 Supplement 1, 2015: Cardiovascular Disease and Vulnerable Plaque Biomechanics. The full contents of the supplement are available online at http://www.biomedical-engineering-online.com/supplements/14/S1

## References

[B1] Kluytmans M van der Grond J van Everdingen KJ Klijn CJM Kappelle LJ Viergever Ma Cerebral Hemodynamics in Relation to Patterns of Collateral Flow Stroke 1999 30 1432 1439 10.1161/01.STR.30.7.1432 10390319

[B2] Powers WJ Cerebral hemodynamics in ischemic cerebrovascular disease Ann Neurol 1991 29 231 240 10.1002/ana.410290302 2042939

[B3] Powers WJ Press GA Grubb RL Gado M Raichle ME The effect of hemodynamically significant carotid artery disease on the hemodynamic status of the cerebral circulation Ann Intern Med 1987 106 27 34 10.7326/0003-4819-106-1-27 3491558

[B4] Schomer DF Marks MP Steinberg GK Johnstone IM Boothroyd DB Ross MR Pelc NJ Enzmann DR The anatomy of the posterior communicating artery as a risk factor for ischemic cerebral infarction N Engl J Med 1994 330 1565 1570 10.1056/NEJM199406023302204 8177246

[B5] Eftekhar B Dadmehr M Ansari S Ghodsi M Nazparvar B Ketabchi E Are the distributions of variations of circle of Willis different in different populations?-Results of an anatomical study and review of literature BMC Neurol 2006 6 22 10.1186/1471-2377-6-22 PMC1543654 16796761

[B6] Amirjamshidi A Mahmoodi R Hashemi SM Variations in the Anatomy of the Willis' circle: A 3-year cross-sectional study from Iran (2006-2009). Are the distributions of variations of circle of Willis different in different populations? Result of an anatomical study and review of literature Surg Neurol Int 2013 4 65 10.4103/2152-7806.112185 23772335 PMC3680999

[B7] Vrselja Z Brkic H Mrdenovic S Radic R Curic G Function of circle of Willis J Cereb Blood Flow Metab 2014 34 578 584 10.1038/jcbfm.2014.7 24473483 PMC3982101

[B8] Lippert H Pabst R Arterial Variations in Man:classification and Frequency 1985 Munich: Springer

[B9] Schneider PA Ringelstein EB Rossman ME Dilley RB Sobel DF Otis SM Bernstein EF Importance of cerebral collateral pathways during carotid endarterectomy Stroke 1988 19 1328 1334 10.1161/01.STR.19.11.1328 3055440

[B10] Baumgartner RW Intracranial stenoses and occlusions, and circle of willis collaterals Front Neurol Neurosci 2006 21 117 126 17290131 10.1159/000092394

[B11] Hoksbergen AWJ Legemate DA Csiba L Csáti G Síró P Fülesdi B Absent collateral function of the circle of Willis as risk factor for ischemic stroke Cerebrovasc Dis 2003 16 191 198 10.1159/000071115 12865604

[B12] Chaudhuri R Padayachee TS Lewis RR Gosling RG Cox TC Non-invasive assessment of the Circle of Willis using transcranial pulsed Doppler ultrasound with angiographic correlation Clin Radiol 1992 46 193 197 10.1016/S0009-9260(05)80444-1 1395425

[B13] Hoksbergen AW Legemate DA Ubbink DT Jacobs MJ Collateral variations in circle of willis in atherosclerotic population assessed by means of transcranial color-coded duplex ultrasonography Stroke 2000 31 1656 1660 10.1161/01.STR.31.7.1656 10884469

[B14] Hoksbergen AW Legemate DA Ubbink DT de Vos HJ Jacobs NJ Influence of the collateral function of the circle of Willis on hemispherical perfusion during carotid occlusion as assessed by transcranial colour-coded duplex ultrasonography Eur J Vasc Endovasc Surg 1999 17 486 492 10.1053/ejvs.1999.0824 10375484

[B15] Miralles M Dolz JL Cotillas J Aldoma J Santiso MA Gimenez A Capdevila A Cairols MA The role of the circle of Willis in carotid occlusion: Assessment with phase contrast MR angiography and transcranial duplex Eur J Vasc Endovasc Surg 1995 10 424 430 10.1016/S1078-5884(05)80164-9 7489210

[B16] Hoksbergen AW Fülesdi B Legemate DA Csiba L Collateral configuration of the circle of Willis: transcranial color-coded duplex ultrasonography and comparison with postmortem anatomy Stroke 2000 31 1346 1351 10.1161/01.STR.31.6.1346 10835455

[B17] Hoksbergen AW Majoie CBL Hulsmans FJ Legemate DA Assessment of the collateral function of the circle of Willis: three-dimensional time-of-flight MR angiography compared with transcranial color-coded duplex sonography AJNR Am J Neuroradiol 2003 24 456 462 12637297 PMC7973617

[B18] Maltezos CK Papanas N Papas TT Georgiadis GS Dragoumanis CK Marakis J Maltezos E Lazarides MK Changes in blood flow of anterior and middle cerebral arteries following carotid endarterectomy: a transcranial Doppler study Vasc Endovascular Surg 2007 41 389 396 10.1177/1538574407302850 17942853

[B19] Anzola GP Gasparotti R Magoni M Prandini F Transcranial Doppler Sonography and Magnetic Resonance Angiography in the Assessment of Collateral Hemispheric Flow in Patients With Carotid Artery Disease Stroke 1995 26 214 217 10.1161/01.STR.26.2.214 7831690

[B20] Muller M Hermes M Bruckmann H Schimrigk K Transcranial Doppler ultrasound in the evaluation of collateral blood flow in patients with internal carotid artery occlusion: correlation with cerebral angiography AJNR Am J Neuroradiol 1995 16 195 202 7900593 PMC8337692

[B21] Krabbe-Hartkamp M Van der Grond J Circle of Willis: morphologic variation on three-dimensional time-of-flight MR angiograms Radiology 1998 207 103 111 10.1148/radiology.207.1.9530305 9530305

[B22] Pennekamp CWA van Laar PJ Hendrikse J den Ruijter HM Bots ML van der Worp HB Kappelle LJ Buhre WF Bleys RLAW Moll FL de Borst GJ Incompleteness of the circle of Willis is related to EEG-based shunting during carotid endarterectomy Eur J Vasc Endovasc Surg 2013 46 631 637 10.1016/j.ejvs.2013.09.007 24091095

[B23] Ito K Sasaki M Kobayashi M Ogasawara K Nishihara T Takahashi T Natori T Uwano I Yamashita F Kudo K Noninvasive Evaluation of Collateral Blood Flow through Circle of Willis in Cervical Carotid Stenosis Using Selective Magnetic Resonance Angiography J Stroke Cerebrovasc Dis 2014 23 1019 1023 10.1016/j.jstrokecerebrovasdis.2013.08.018 24103664

[B24] Shaban A Albright KC Boehme AK Martin-Schild S Circle of Willis Variants: Fetal PCA Stroke Res Treat 2013 2013 105937 23577277 10.1155/2013/105937 PMC3618940

[B25] Cebral JR Putman CM Alley MT Hope T Bammer R Calamante F Hemodynamics in Normal Cerebral Arteries: Qualitative Comparison of 4D Phase-Contrast Magnetic Resonance and Image-Based Computational Fluid Dynamics J Eng Math 2009 64 367 378 10.1007/s10665-009-9266-2 19684874 PMC2726749

[B26] Ricci M Cornacchiola V Pigliautile M Ercolani S Mecocci P Fetal variant of circle of the Willis and bilateral symmetrical parietal stroke Neurol Sci 2012 33 309 311 10.1007/s10072-011-0681-6 21732065

[B27] Arjal RK Zhu T Zhou Y The study of fetal-type posterior cerebral circulation on multislice CT angiography and its influence on cerebral ischemic strokes Clin Imaging 2014 38 221 225 10.1016/j.clinimag.2014.01.007 24602416

[B28] Mull M Schwarz M Thron A Cerebral hemispheric low-flow infarcts in arterial occlusive disease. Lesion patterns and angiomorphological conditions Stroke 1997 28 118 123 10.1161/01.STR.28.1.118 8996499

[B29] Bladin CF Colchester ACF Hawkes DJ Seifalian AM Iqbal N Hardingham CR Morphological and Hemodynamic Assessments of Carotid Stenosis Using Quantitative Digital Subtraction Angiography Stroke 1996 27 1672 1678 10.1161/01.STR.27.9.1672 8784147

[B30] Alastruey J Parker KH Peiró J Byrd SM Sherwin SJ Modelling the circle of Willis to assess the effects of anatomical variations and occlusions on cerebral flows J Biomech 2007 40 1794 1805 10.1016/j.jbiomech.2006.07.008 17045276

[B31] Moore SM Moorhead KT Chase JG David T Fink J One-dimensional and three-dimensional models of cerebrovascular flow J Biomech Eng 2005 127 440 449 10.1115/1.1894350 16060350

[B32] Zhang C Wang L Li X Li S Pu F Fan Y Li D Modeling the circle of Willis to assess the effect of anatomical variations on the development of unilateral internal carotid artery stenosis Biomed Mater Eng 2014 24 491 499 24211932 10.3233/BME-130835

[B33] DeVault K Gremaud P Novak V Blood flow in the circle of Willis: Modeling and calibration Multiscale Model Simul 2008 7 888 909 10.1137/07070231X 19043621 PMC2587352

[B34] Grinberg L Cheever E Anor T Madsen JR Karniadakis GE Modeling blood flow circulation in intracranial arterial networks: a comparative 3D/1D simulation study Ann Biomed Eng 2011 39 297 309 10.1007/s10439-010-0132-1 20661645

[B35] Abdi M Karimi A Navidbakhsh M Rahmati M Hassani K Razmkon A Modeling the Circle of Willis Using Electrical Analogy Method under both Normal and Pathological Circumstances J Biomed Phys Eng 2013 3 45 56 25505747 PMC4204494

[B36] Cassot F Zagzoule M Marc-Vergnes JP Hemodynamic role of the circle of Willis in stenoses of internal carotid arteries. An analytical solution of a linear model J Biomech 2000 33 395 405 10.1016/S0021-9290(99)00193-1 10768388

[B37] Ferrandez A David T Brown MD Numerical models of auto-regulation and blood flow in the cerebral circulation Comput Methods Biomech Biomed Engin 2002 5 7 19 10.1080/10255840290032171 12186730

[B38] Ferrández A David T Bamford J Scott J Guthrie A Computational Models of Blood Flow in the Circle of Willis Comput Methods Biomech Biomed Engin 2000 4 1 26 11264859 10.1080/10255840008907996

[B39] Long Q Luppi L König CS Rinaldo V Das SK Study of the collateral capacity of the circle of Willis of patients with severe carotid artery stenosis by 3D computational modeling J Biomech 2008 41 2735 2742 10.1016/j.jbiomech.2008.06.006 18674765

[B40] Reorowicz P Obidowski D Klosinski P Szubert W Stefanczyk L Jozwik K Numerical simulations of the blood flow in the patient-specific arterial cerebral circle region J Biomech 2014 47 1642 1651 10.1016/j.jbiomech.2014.02.039 24674598

[B41] Oshima M Torii R Tokuda S Yamada S Koizumi A Patient-Specific Modeling and Multi-Scale Blood Simulation for Computational Hemodynamic Study on the Human Cerebrovascular System Curr Pharm Biotechnol 2012 13 2153 2165 10.2174/138920112802502105 22335478

[B42] Alnaes MS Isaksen J Mardal KA Romner B Morgan MK Ingebrigtsen T Computation of hemodynamics in the circle of Willis Stroke 2007 38 2500 2505 10.1161/STROKEAHA.107.482471 17673714

[B43] Cebral JR Castro MA Soto O Löhner R Alperin N Blood-flow models of the circle of Willis from magnetic resonance data J Eng Math 2003 47 369 386

[B44] Cieslicki K Ciesla D Investigations of flow and pressure distributions in physical model of the circle of Willis J Biomech 2005 38 2302 2310 10.1016/j.jbiomech.2004.07.038 16154418

[B45] Fahy P McCarthy P Sultan S Hynes N Delassus P Morris L An experimental investigation of the hemodynamic variations due to aplastic vessels within three-dimensional phantom models of the circle of Willis Ann Biomed Eng 2014 42 123 138 10.1007/s10439-013-0905-4 24018609

[B46] Ujiie H Liepsch DW Goetz M Yamaguchi R Yonetani H Takakura K Hemodynamic study of the anterior communicating artery Stroke 1996 27 2086 2093 discussion 2094 10.1161/01.STR.27.11.2086 8898821

[B47] Moore S David T Chase JG Arnold J Fink J 3D models of blood flow in the cerebral vasculature J Biomech 2006 39 1454 1463 10.1016/j.jbiomech.2005.04.005 15953607

[B48] Alastruey J Parker KH Peiró J Byrd SM Sherwin SJ Modelling the circle of Willis to assess the effects of anatomical variations and occlusions on cerebral flows J Biomech 2007 40 1794 1805 10.1016/j.jbiomech.2006.07.008 17045276

[B49] Jou LD Lee DH Mawad ME Cross-flow at the anterior communicating artery and its implication in cerebral aneurysm formation J Biomech 2010 43 2189 2195 10.1016/j.jbiomech.2010.03.039 20447636

[B50] Hartkamp MJ van der Grond J van Everdingen KJ Hillen B Mali WPTM Circle of Willis Collateral Flow Investigated by Magnetic Resonance Angiography Stroke 1999 30 2671 2678 10.1161/01.STR.30.12.2671 10582995

[B51] Hendrikse J van Raamt a F van der Graaf Y Mali WPTM van der Grond J Distribution of cerebral blood flow in the circle of Willis Radiology 2005 235 184 189 10.1148/radiol.2351031799 15749975

[B52] Hillen B Gaasbeek T Hoogstraten HW A mathematical model of the flow in the posterior communicating arteries J Biomech 1982 15 441 448 10.1016/0021-9290(82)90080-X 7118958

[B53] Van de Vosse FN Stergiopulos N Pulse Wave Propagation in the Arterial Tree Annu Rev Fluid Mech 2011 43 467 499 10.1146/annurev-fluid-122109-160730

[B54] Steinman DA Ethier CR The effect of wall distensibility on flow in a two-dimensional end-to-side anastomosis J Biomech Eng 1994 116 294 301 10.1115/1.2895733 7799630

[B55] Xu XY Collins MW Jones CJ Flow studies in canine artery bifurcations using a numerical simulation method J Biomech Eng 1992 114 504 511 10.1115/1.2894102 1487903

[B56] Liebeskind DS Collateral circulation Stroke 2003 34 2279 2284 10.1161/01.STR.0000086465.41263.06 12881609

[B57] Enzmann DR Ross MR Marks MP Pelc NJ Blood flow in major cerebral arteries measured by phase-contrast cine MR AJNR Am J Neuroradiol 1994 15 123 129 8141043 PMC8332086

[B58] Van Ooij P Zwanenburg JJM Visser F Majoie CB Hendrikse J Nederveen AJ Quantification and Visualization of Flow in the Circle of Willis: Time-Resolved Three-Dimensional Phase Contrast MRI at 7 T Compared with 3 T Magn Reson Med 2013 69 868 876 10.1002/mrm.24317 22618854

[B59] Zhao M Amin-Hanjani S Ruland S Curcio AP Ostergren L Charbel FT Regional Cerebral Blood Flow Using Quantitative MR Angiography Am J Neuroradiol 2007 28 1470 1473 10.3174/ajnr.A0582 17846193 PMC8134363

